# Comparison of hand-sewn and stapled anastomoses in surgeries of gastrointestinal tumors based on clinical practice of China

**DOI:** 10.1186/1477-7819-12-292

**Published:** 2014-09-21

**Authors:** Bin-wei Liu, Yang Liu, Jun-ru Liu, Zhong-xu Feng

**Affiliations:** Department of General Surgery, The First Hospital of Qinhuangdao, Qinhuangdao, Hebei China; Department of Endocrinology, The First Hospital of Qinhuangdao, Qinhuangdao, Hebei China

**Keywords:** Stapler, Gastrocolorectal tumor, Anastomosis, Surgery

## Abstract

**Background:**

There is a lack of studies comparing stapled suturing and hand-sewn suturing in the surgeries of gastrointestinal tumors based on the clinical practice of Chinese surgeons.

**Methods:**

Data were retrospectively collected from 499 patients who underwent surgery to remove gastrointestinal tumors from January 2008 to December 2009. The patients were divided into two groups according to the method of digestive tract reconstruction: 296 patients received stapled suturing and 203 patients received hand-sewn suturing. The operation time, postoperative hospital stay, postoperative recovery and complications of the patients were evaluated and compared between the two groups.

**Results:**

The stapling procedure took shorter operative time compared to the hand-sewn procedure for gastric carcinoma, colorectal cancer and esophageal carcinoma (*P* < 0.05). There was no significant difference between the two groups in postoperative hospital stay (*P* > 0.05). Patients receiving stapled suturing also showed shorter recovery for gastric cancer, colorectal cancer, and shorter time to recovery of normal gastrocolorectal motility compared with patients in the hand-sewn group (*P* < 0.05). However, there was no difference between the two groups in terms of normal time to commencing liquid diet for esophageal cancer patients (*P* > 0.05). We also found that the stapled procedure showed a lower incidence of anastomotic leakage, anastomotic hemorrhage and stump leakage in treating colorectal cancer or gastric carcinoma compared with the hand-sewn procedure (*P* < 0.05).

**Conclusions:**

Application of the stapler in treating gastrointestinal tumors demonstrated better effects on patients in terms of surgical operation time, recovery time to normal functions, and occurrence of complications compared to hand-sewn anastomosis, especially in gastric carcinoma and colorectal cancer.

## Background

Surgical excision was previously considered as the main method for treating gastrointestinal (GI) tumors. Anastomotic procedure is one of the key factors determining surgical success. Hand-sewn and stapled sutures comprise the major anastomotic methods in clinical practice of GI surgeries.

Staplers were originally developed to address the perceived problem of patency (security against leakage of blood or bowel contents) in anastomoses in particular [1]. After the introduction of stapled colorectal anastomosis in the 1980s, both conventional hand-sewn and stapled anastomosis have become prevalent. So far, no defined indications have been defined and most surgeons apply both techniques in clinical practice. Multiple reports reviewed available data on the trials evaluating the two techniques in colorectal surgeries and some of these results are conflicting. A systemic review evaluating 1,233 patients showed no significant difference in mortality, anastomotic leak, strictures or re-operation between stapled and hand-sewn colorectal anastomosis {Lustosa, 2001 #152} [2]. However, another comprehensive review evaluating 1,125 ileocolic participants showed stapled anastomosis was associated with fewer leaks than hand-sewn anastomosis although there was no difference for stricture formation, anastomotic hemorrhage, anastomotic time, re-operation rate, mortality rate, or intra-abdominal abscess formation [3].

There has been a lack of studies comparing superiority in general surgeries of digestive tract tumors between stapled and hand-sewn anastomosis and data based on Chinese patients is scant. Here we evaluated the clinical application value of the stapler suture in tumor surgery for digestive tract tumors by analyzing 499 patients who were treated for tumors of the digestive tract at nine hospitals in China. We aimed to probe the differences in anastomotic effect between stapler and manual suture in treating GI tumors in order to provide scientific basis for reasonable application of stapling based on Chinese clinical data.

## Methods

### Patient selection

The Institutional Review Board of our hospital approved the study, which was conducted in accordance with the ethical principles of the Declaration of Helsinki and consistent with good clinical practices and applicable laws and regulations. All patients provided written informed consent prior to enrollment.

Patients who underwent surgery for GI tumors at hospitals in three provinces between January 2008 and December 2009, were enrolled in the study. GI tumors included gastric carcinoma, colorectal cancer and esophageal carcinoma. Exclusion criteria include: tumors with lymph node metastasis or tumors invading neighboring tissue; unresectable tumors.

### Research methods

The data were collected retrospectively. All the cases were observed for one month after operation and the data relating to recovery were recorded. The following clinical characteristics were compared between the stapled and hand-sewn groups: operation time, postoperative hospital stay, postoperative recovery (time of gastrocolorectal normal motility and time to commencing normal liquid diet) and complications (anastomotic leakage, anastomotic hemorrhage, stump leakage and occurrence of emergency associated with post-surgery-ICU stay).

### Statistics

Database of cases was created using Epidate3.1 (The Epidata Association, Denmark). The data of cases were processed by SPSS 19.0 software (Chicago, IL, USA). Quantitative data were expressed as *x* ± SD and discrete variables were presented using frequency table and descriptive statistical method. Comparisons between the two groups were processed using the Student’s *t*-test, rank sum test (quantitative data) or chi-squared test (categorical data). For all the tests, *P* < 0.05 was considered statistically significant.

## Results

The stapled group consisted of 296 patients, including 187 male and 109 female patients, 239 cases from municipal hospitals and 57 cases from county hospitals. The average age was 59.05 ± 10.18 years. The group of digestive tract anastomosis performed with manual procedure contained 203 cases; 136 male and 67 female patients from 165 municipal hospitals and 38 county hospitals. The average age was 57.50 ± 10.05 years. There were no significant difference between the two groups in gender, hospital composition and age (*P* > 0.05) (gender: *χ*^*2*^ = 0.769, *P =* 0.38; hospital composition: *χ*^*2*^ = 0.027, *P =* 0.88; age: *t* = 1.65, *P* > 0.05). The stapler group consisted of 104 patients with gastric cancer, 128 patients of colorectal cancer and 64 patients with esophageal carcinoma respectively. The hand-sewn group consisted of 74 patients with gastric cancer, 97 patients with colorectal cancer and 32 patients with esophageal carcinoma respectively. Overall, there was no significant difference between the two groups in terms of the cancer composition (*P* > 0.05).

**Figure 1:**
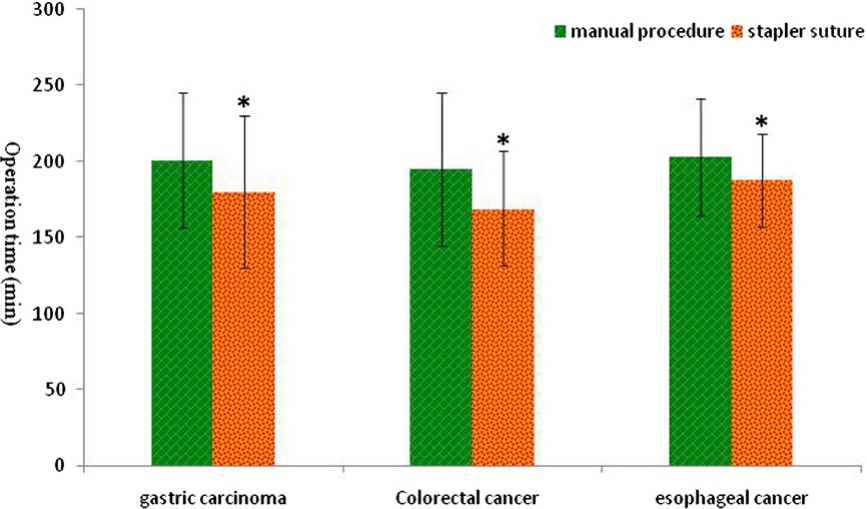
**Comparison of the operation time between manual and stapled anastomosis in the three types of gastrointestinal (GI) tumor.** Values are presented as mean ± standard deviation (), and * indicates the statistical significance (*P* < 0.05).

Firstly we compared the operation time of gastric cancer, colorectal cancer and esophageal carcinoma in the two groups respectively (Figure 1) and the results suggest stapled anastomosis significantly shortened the operation time in the patients for the three types of tumor (*P* < 0.05).

**Figure 2:**
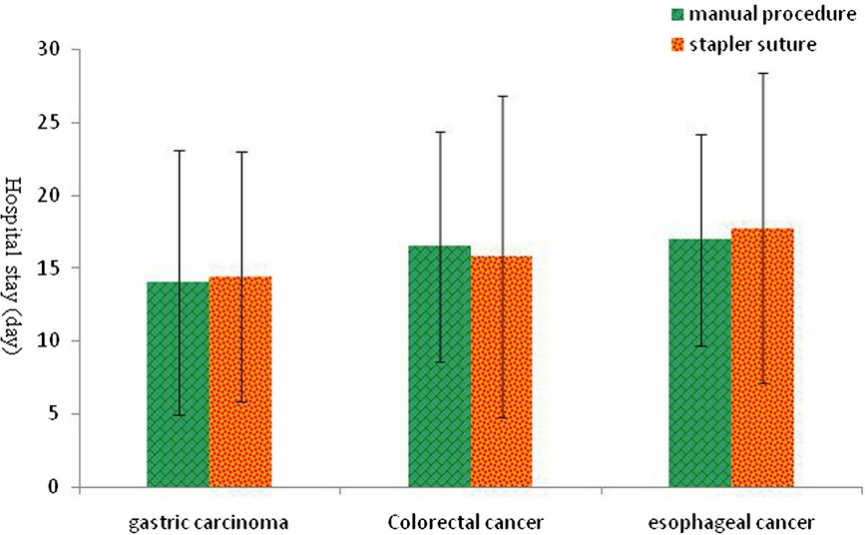
**Comparison of the days of hospital stay between the manual and stapled anastomosis groups after surgery.** Values are presented as mean ± standard deviation (), and * indicates the statistical significance (*P* < 0.05).

We further analyzed the number of days of postoperative hospital stay of the two groups and the results suggested no statistical significant between hand-sewn and stapled procedures for all three types of tumor (*P* > 0.05) (Figure 2).

**Figure 3:**
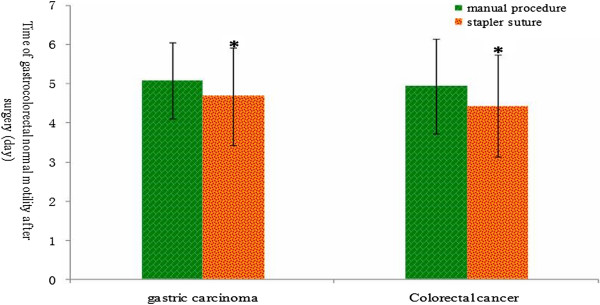
**Comparison of recovery time to normal gastrointestinal (GI) motility between manual and stapled anastomosis after surgery.** Values are presented as mean ± standard deviation (), and * indicates the statistical significance (*P* < 0.05).

To evaluate the recovery of digestive tract functions after surgery, we also compared time of recovery to normal gastrocolorectal motility after surgery between the two groups. The results revealed that the stapled procedure significantly shortened the time of recovery to normal functions of the digestive tract compared to the hand-sewn procedure for gastric carcinoma and colorectal cancer (*P* < 0.05) but not esophageal carcinoma (*P* > 0.05) (Figure 3). For patients with esophageal carcinoma, we evaluated recovery time to commencing normal liquid diet after surgery and it turned out that using the stapled procedure did not alter the recovery time compared to the hand-sewn procedure (Table 1).

**Table 1:** **Comparison of recovery time to commencing normal liquid diet between the manual and stapled anastomosis groups after surgery**

Tumor types	Anastomotic methods	Number of cases	The normal time for liquid diet after surgery (day) ( )	***t-***value	***P-***value
Esophageal cancer	Manual procedure	32	8.38 ± 3.11	0.607	> 0.05
	Stapler suture	64	8.02 ± 1.77		

To evaluated complications after manual and stapled anastomosis, we compared anastomotic leakage, anastomotic hemorrhage, stump leakage and the post-surgery-ICU requirement between the two groups. We found that stapler suturing reduced the incidence of anastomotic leakage for gastric carcinoma and colorectal cancer compared to manual suturing (*P* < 0.05) (Table 2). Stapled procedure also significantly lowered the incidence of anastomotic hemorrhage for the three types of digestive tract tumor (*P* < 0.05). For stump leakage, the stapler suture effectively reduced the occurrence for colorectal cancer (*P* < 0.05), but not for gastric carcinoma (*P* > 0.05) (Table 2). For post-surgery-ICU requirement, there was no difference between the two groups for the three types of tumor (*P* > 0.05) (Table 2).

**Table 2:** **Comparison of complication occurrence between the manual and stapled anastomosis groups after surgery**

Anastomotic methods	Gastric carcinoma					Colorectal cancer					Esophageal cancer			
	S-lE	A-lE ^a^	A-HH ^a^	PS-ICU	Total	S-lE ^a^	A-lE ^a^	A-HH ^a^	PS-ICU	Total	A-lE	A-HH ^a^	PS-ICU	Total
Manual procedure	7	11	12	10	74	15	14	16	18	97	4	6	7	32
Stapler suture	4	6	6	8	104	8	8	10	23	128	4	4	8	64

^a^Indicates *P* < 0.05. *The Abbreviations*: *S-lE* (stump leakage), *A-lE* (anastomotic leakage), *A-HH* (anastomotic hemorrhage), and *PS-ICU* (post-surgery-ICU).

## Discussion

GI tumors represent a serious threat to the health of Chinese people. GI tumors, such as gastric cancer, liver cancer and esophageal cancer, continue to rank as the top five cancers during the past three decades in China [4]. China is one of the countries with the highest incidence of gastric cancer, which accounts for over 40% of all new gastric cancer cases in the world [5]. For esophageal cancer in China, the crude mortality rate in 2004 to 2005 was 15.2/100,000, which represented 11.2% of all cancer deaths and ranked as the fourth most common cause of cancer death [6]. The incidence of colorectal cancer in China is generally lower than that in western countries, but has increased in recent years particularly in the more developed areas [7]. At present, surgery remains the major first-line treatment for these three tumors. The surgical treatment for digestive tract tumors normally involves partial or total organ removal, surrounding lymph node clearance and post-resection digestive tract reconstruction. Anastomosis is generally performed for the surgery treating these three types of GI tract tumor. An ideal anastomosis should fulfill the following criteria: it must be well vascularized, safe, tension-free and spillage from the operation field should be avoided [8]. The manual procedure has been used in tract anastomosis for a long time, but stapler suturing has been increasingly used as an anastomotic method in digestive tract surgery in the past few years [9, 10].

Our results demonstrated that stapler suturing shortened the operation time compared to conventional hand-sewn suturing for all three types of digestive tract tumor. Shortening the operation time means reducing surgical trauma and intra-operative blood loss and also abating local infection and reducing the chance of surgical complications. However, the superiority of stapling did not extend to postoperative hospital stay, which is consistent with the previous reports that postoperative hospital stay time showed no difference between the patients who received stapler suturing and manual procedure after gastrectomy [9, 11]. However, another study showed that stapling anastomosis shortens postoperative hospital stay in the patients with stomach and esophageal tumors [12]. This discrepancy could be due to stapling treatment and the empirical level of surgical operators.

When comparing the functional postoperative recovery between the two groups, the time to normal gastrocolorectal motility after surgery was shorter in the stapler group compared to the manual group (*P* < 0.05) indicating the superiority of stapling in postoperative recovery. This could be explained by minor surgical trauma around stapler anastomosis and the contraposition of anastomotic tissues, avoiding damage to the gastric mucosa from the cutting thread [11, 13].

We also demonstrated that stapler suturing is superior to the manual method in reducing the incidence of anastomotic leakage for gastric carcinoma and colorectal cancer, and the incidence of anastomotic hemorrhage for gastric carcinoma, colorectal cancer and esophageal cancer. The stapler suture also effectively lowered the occurrence of stump leakage for colorectal cancer compared to the hand-sewn method. Although major meta-analysis and comprehensive reviews suggest no significant difference was found in in terms of restoration of intestinal function, postoperative hospital stay and postoperative complications, it is recognized that the stapler method generally shortens the total operating time and provides better access to difficult-to-reach areas [14–16]. We obtained similar results in operative time and hospital stay but showed superiority of stapled method in reducing several complications. Consistent with our findings, a few studies did show better outcomes of stapler anastomosis in preventing occurrence of complications. For instance, stapled esophagogastric anastomosis could prevent stricture formation more effectively than hand-sewn, without increasing gastroesophageal reflux [17, 18]. Another study also revealed the side-to-side stapled technique is conducive in decreasing complications of postoperative dysphagia and is helpful for improving pharyngesophageal and anastomotic menometric function [19]. Interestingly, the stapled method had a higher incidence of anastomotic stricture according to another report [20].

There is a relative lack of reports on the functional recovery of the organs after surgery in patients with digestive tract cancers. Our results did show that the stapler method significantly shortened the time to GI normal motility after surgery for both gastric and intestinal cancer patients. However, the stapler method was not superior to conventional methods in terms of the normal time to commencing liquid diet after surgery for esophageal cancer patients.

The stapled procedure could be translated to the benefits of functional recovery or reduced complications via: consistent space of anastomosis; tight closure of anastomotic nails, type “B” cross-stich using titanium nails [12]; the consistency of anastomosis and cutting may reduce the chance of infection due to manual operation and decrease the risk of pulling and clamping the jejunum, thus reducing jejunal damage.

## Conclusions

Overall, our current study based on the clinical practice of Chinese physicians showed stapled anastomosis to be superior to the conventional hand-sewn method in terms of operative time, postoperative organ functional recovery time and the incidence of several complications, especially in gastric and colorectal cancers. The findings were somewhat inconsistent with most reported, which concluded that stapled and hand-sewn sutures had similar outcome in terms of complications. Nevertheless, our novel results indicating superiority of stapled anastomosis in term of recovery time of gastrocolorectal normal motility suggest the stapled is still a preferable choice to the hand-sewn method in gastric and colorectal cancers given the other advantages of stapler anastomosis, such as shorter operative time.

## Abbreviations

GI: gastrointestinal
S-lE: stump leakage
A-lE: anastomotic leakage
A-HH: anastomotic hemorrhage
PS-ICU: post-surgery-ICU.
